# Liquid-Assisted Redox Synthesis Enables High-Yield Metastable Tetragonal Spinel Oxides

**DOI:** 10.1021/acsmaterialsau.6c00107

**Published:** 2026-07-22

**Authors:** Yanita Devi, Chun-Hu Chen

**Affiliations:** † Department of Chemistry, National Sun Yat-sen University, Kaohsiung, Taiwan 80424; ‡ Green Hydrogen Research Center, 34874 National Sun Yat-sen University, Kaohsiung, Taiwan 80424

**Keywords:** Redox-precipitation kinetics, Metastable phase synthesis, Tetragonal spinel oxide, Cobalt−manganese oxides, oxygen evolution reaction, Jahn−Teller distortion, trace amount of water

## Abstract

Controlling phase selectivity for especially metastable multimetal oxides remains a central challenge, even though they are attractive for catalysis and energy conversion applications. The conventional synthesis method, like aqueous redox-precipitation, typically uses dilute reactant solutions, in which thermodynamic equilibrium favors stable products, limiting the selective synthesis of metastable phases. Here, we demonstrate a water-assisted reaction that plays a dual and decisive role in liquid-assisted redox synthesis (LRS) to obtain a metastable tetragonal cobalt–manganese spinel (CMO), rather than the readily formed cubic spinel. Limited initial hydration (0.5–1.5 mL) creates a highly concentrated microenvironment that enhances local acidity and shifts redox toward Mn^3+^ formation rather than Mn^4+^. Dehydration at 160 °C enables the incorporation of unstable Mn^3+^ into the solid lattice, resulting in high selectivity and phase purity toward the metastable tetragonal spinel. A low liquid-to-solid ratio (η = 0.27–0.81 μL mg^–1^) coupled with elevated dehydration temperatures (up to 200 °C) enabled the selective formation of metastable tetragonal spinel. Increasing the initial water content (≥3.0 mL) without complete water dehydration further restores bulk-solution-like behavior, promotes Mn^3+^/Mn^4+^ equilibration, and yields the thermodynamic cubic spinel. The optimized condition delivers 88% yield (∼27× higher than conventional aqueous ARP, 3.2%), and the tetragonal phase exhibits enhanced alkaline (oxygen evolution reaction) OER activity relative to the cubic analog.

## Graphical abstract

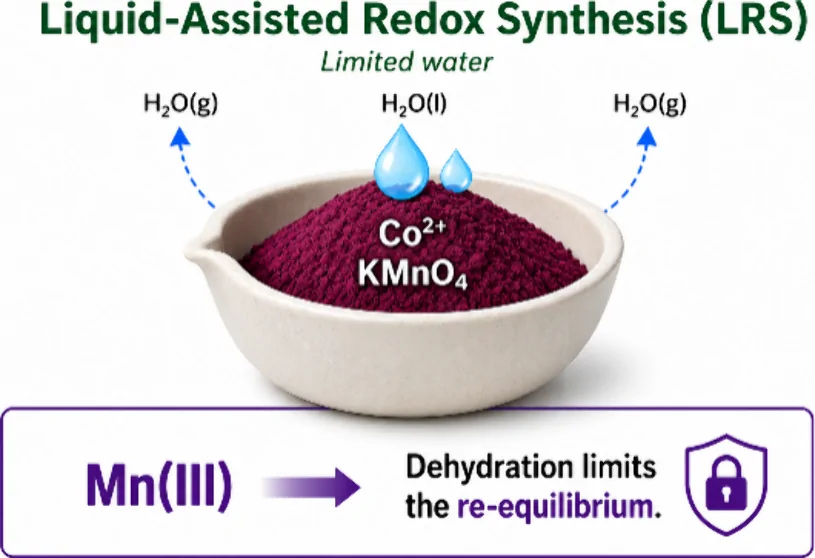

## 1 Introduction

Metastable multimetal oxides often exhibit distinct electronic structures, local coordination environments, and defect chemistries relative to those of their thermodynamic counterparts, and these features can translate into enhanced functional performance.^1,2^ However, synthesizing these metastable states remains challenging because they tend to relax toward equilibrium during nucleation and growth.^3–5^ Existing routes frequently rely on specialized ligands/coordination environments or on stringent processing windows, which complicate synthesis and often limit yields and scalability.^6,7^ As a result, achieving both high phase selectivity and scalable yield remains a central challenge in materials synthesis.

Spinel oxides exemplify this challenge. Thermodynamically favored cubic spinels are widely accessible and readily produced at scale.^8^ In contrast, their distorted derivatives (e.g., tetragonal spinels) are typically metastable and therefore difficult to isolate as phase-pure products.^9,10^ In practice, changes in reaction environment and precipitation/growth conditions often generate mixed-phase products, making it difficult to isolate as phase-pure products^11,12^ In particular, the formation of tetragonal spinel structures is closely associated with the presence of Jahn–Teller-active Mn^3+^ species, which are intrinsically unstable in aqueous environments and readily undergo disproportionation toward Mn^2+^ and Mn^4+^.^13^ Therefore, controlling both the oxidation state and the reaction environment during synthesis is essential for accessing metastable spinel phases.

A range of approaches have been explored to access metastable phases, including ligand-based stabilization of specific oxidation states (e.g., Mn^3+^),^13^ high-temperature treatments under controlled oxygen environment,^14^ and solid-state/electrochemical trapping pathways that generate Mn^3+^-containing products without requiring free Mn^3+^ in solution.^15^ Despite success in selected systems, these methods often involve harsh conditions, heavy use of structure-directing agents, or narrow process windows. Thus, the products are still suffering from poor phase selectivity and low production yields. Compared with the conventional aqueous redox-precipitation methods, that favor thermodynamic equilibration and limit access to metastable states, recent studies have shown that metastable oxide phase selection is often governed by nonequilibrium crystallization pathways, like dehydration processes to suppress equilibration and stabilize nonequilibrium phases.^16^ The concept of accessing metastable materials through kinetic control can enable the formation and stabilization of these phases that are inaccessible under equilibrium synthesis routes.^2^

Solvent-free (solid-state) synthesis relies on heterogeneous particle collisions to drive solid-state reactions but often suffers from incomplete mixing and multiphase impurity formation.^17–19^ The subsequent introduction of a small amount of liquid during precursor mixing enhances molecular and ionic mobility, promoting a more homogeneous reactant distribution and reaction pathways.^20–22^ This results in a higher collision frequency, leading to an improved homogeneity and more uniform redox chemistry during material formation. This concept aligns naturally with the acidic redox-assisted precipitation (ARP) platform, in which direct redox interactions between metal-containing oxidants and metal cations spontaneously generate mixed-metal oxide or oxyhydroxide precipitates under acidic conditions.^23–25^ ARP shares a similar reaction principle to that of acidic redox-assisted deposition (ARD), a versatile strategy for producing metal oxide thin films under mild conditions.^26^ However, unlike ARD, where the redox reaction occurs preferentially at the substrate surface to generate adherent thin films, ARP focuses on recovering the precipitated products formed throughout the reaction medium. The presence of bulk solvent in conventional ARP systems maintains a fully solvated environment, allowing continued redox equilibration and favoring thermodynamically stable phases.

By combining the advantages of both approaches, liquid-assisted redox synthesis (LRS) uses a limited amount of water as the initial dissolution medium, followed by dehydration during heating, which further shortens the lifetime of the hydrated state. This water dehydration suppresses further solution-mediated equilibration and promotes kinetic trapping of metastable oxide phases. As a result, the oxidation state and local coordination environment are effectively “frozen” before structural relaxation can occur, enabling stabilization of the tetragonal spinel structure.

Herein, we propose that phase selection in liquid-assisted redox synthesis (LRS) is governed by changes in water content during heating, where water initially facilitates redox interactions and is subsequently removed as the reaction progresses. This work highlights that phase selection is governed by minimum water addition and complete dehydration, providing a strategy for accessing cobalt manganese metastable oxides. The selective formation of a metastable tetragonal CMO spinel gives a high yield (86.3 ± 3.0%), representing a major improvement over conventional aqueous ARP (3.2 ± 1.8%). XAS further confirms an enhanced Jahn–Teller distortion of the MnO_6_ octahedra under low-liquid conditions, providing a structural signature of the metastable tetragonal phase. Finally, preliminary electrochemical measurements indicate that the tetragonal CMO exhibits higher (oxygen evolution reaction) OER activity than its cubic analog, highlighting functional consequences of the kinetically trapped structure.

## 2 Results and Discussions

### 2.1 Phase Formation and Yield Optimization of Cobalt Manganese Oxide (CMO)

To establish a limited liquid-assisted redox-precipitation regime for metastable spinel formation, we synthesized cobalt–manganese oxide using only trace water, thereby maintaining high local reactant activities while enabling sufficient ionic mobility. We first optimized the reaction condition by varying the temperature and combining it with initial water addition. This parameter screen provides the experimental basis for the optimized thermal-energy-dependent phase structure. Further, the variation in the existing water that remains in the system during the heating process was also studied to understand how phase selection varies with dehydration. For clarity, the samples are denoted as T-x, where T represents the synthesis temperature (in °C) and x denotes the amount of water added (in mL). For instance, a sample synthesized at 160 °C with 1.0 mL of water is labeled as 160–1.0. Calcined samples (500 °C, 2 h) are denoted as T–x–cal. Detailed experimental procedures are provided in the Experimental Section. To quantitatively describe the reaction environment and facilitate comparison with other liquid-assisted synthesis methods, the liquid-to-solid ratio (η) corresponding to each water addition condition was calculated and is summarized in Table S1 of the Supporting Information.

Powder X-ray diffraction (XRD) of the calcined products (Figure 1) was used to evaluate phase evolution as a function of temperature at fixed trace-water loading, followed by a targeted study at the optimized temperature. At lower temperatures (90–0.5-cal and 120–0.5-cal), only the major reflections of tetragonal spinel CMO (e.g., (220), (311), and (440); JCPDS 18-0408) are apparent, together with an additional feature near 2θ ≈ 27.9°, most likely due to incomplete crystallization. Upon increasing the synthesis temperature to ≥160 °C (160–0.5-cal and 200–0.5-cal), the diffraction pattern becomes fully consistent with tetragonal spinel CMO (JCPDS 18-0408) without detectable impurity phases. The emergence of diagnostic tetragonal reflections (e.g., (202), (113), and (404)), absent at lower temperature, together with the expected (111), (220), (311), (400), and (440) peaks, confirms formation of the tetragonal spinel (space group *I*4_1_/*amd*). These results identify trace water of the LRS method at ≥160 °C as a regime that selectively yields the metastable tetragonal spinel.

**Figure 1:**
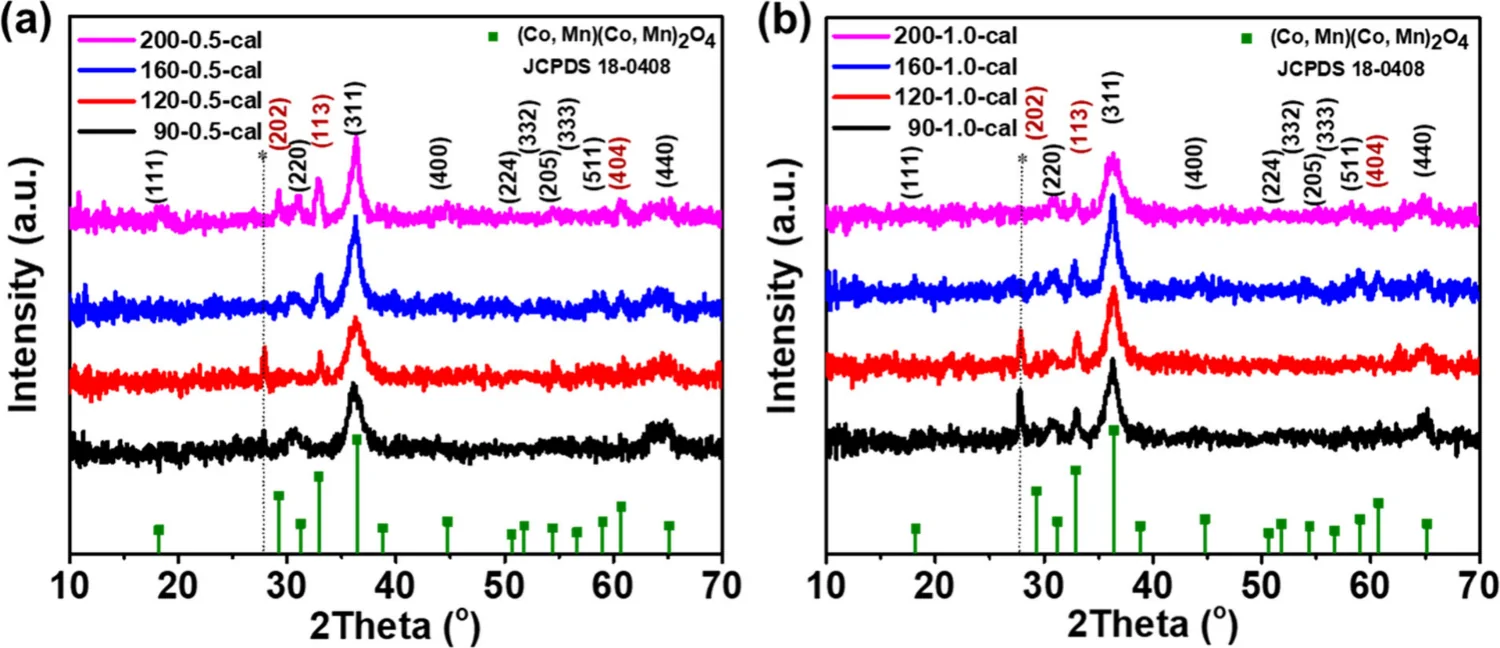
Powder X-ray diffraction (PXRD) patterns of the LRS system products synthesized at 90–200 °C with fixed water additions of (a) 0.5 mL and (b) 1.0 mL, followed by calcination at 500 °C. The dotted line at 2θ = 27.9° (asterisk) marks an unidentified reflection observed in the low-temperature products. The reference pattern for the tetragonal spinel phase (JCPDS 18-0408) is included for comparison.

To examine the correlation of phase selectivity to modest changes in initial water addition, we increased the water loading to 1.0 mL (Figure 1b). The same trend is observed: the impure-phase feature at ∼27.9° persists at 90–120 °C, whereas at 160–200 °C a single tetragonal spinel phase is obtained. Accordingly, 160 °C was selected as the standard condition in subsequent experiments. In the next section, we systematically vary the initial added water volume to establish how the initial water-assisted and water content during the heating reaction govern (i) tetragonal versus cubic spinel preference and (ii) overall conversion/yield in the LRS method.

### 2.2 Redox-Driven Formation of Tetragonal Spinel CMO under Controlled Water Loading

We next study the role of initial water addition in the LRS system and examine how changes in the reaction environment govern phase selectivity in permanganate (MnO_4_^–^)-driven redox-precipitation with Co^2+^. The addition of initial water promotes more effective interparticle collisions than the pure solid-state synthesis. In the limited amount of water added, the high precursor concentration region shifts the chemical equilibrium fully in favor of the products, thereby increasing the product yield.^27^ Accordingly, changing the initial water volume is expected to vary the initial precursor concentration and the reaction environment (like pH) and, combined with the dehydration during the heating reaction, further control the preferred metastable phase.

To test this hypothesis, XRD analysis was performed on both uncalcined and calcined products prepared at 160 °C with water additions ranging from 0.5 to 10.0 mL (Figure 2). A no-water control (160–0-cal) was also included as a solvent-free reference. In the absence of added water, the product is phase-mixed (Figure S1), comprising tetragonal spinel (Co,Mn)­(Co,Mn)_2_O_4_ (JCPDS 18-0408) and a tunnel-type hollandite phase, K^+^(MnO_2_)_8_ (JCPDS 29-1020). This outcome is due to insufficient transport of ions, in which limited diffusion prevents complete redox conversion and thus favors impurity formation. Similar behavior has been reported in other solid-state redox systems employing permanganate-based oxidants without any solvent mediation. For example, the reaction between KMnO_4_ and Co^(II)^ under solvent-free conditions yields a mixed phase of MnO_2_–CoOOH and poorly crystallized products,^28^ as observed with the solid-state reaction of KMnO_4_, which gives a mixed-phase product containing both α- and δ-MnO_2_ phases.^12^ Thus, introducing a limited amount of water is essential to assist with precursor dissolution and enables controlled phase evolution in the LRS system.

**Figure 2:**
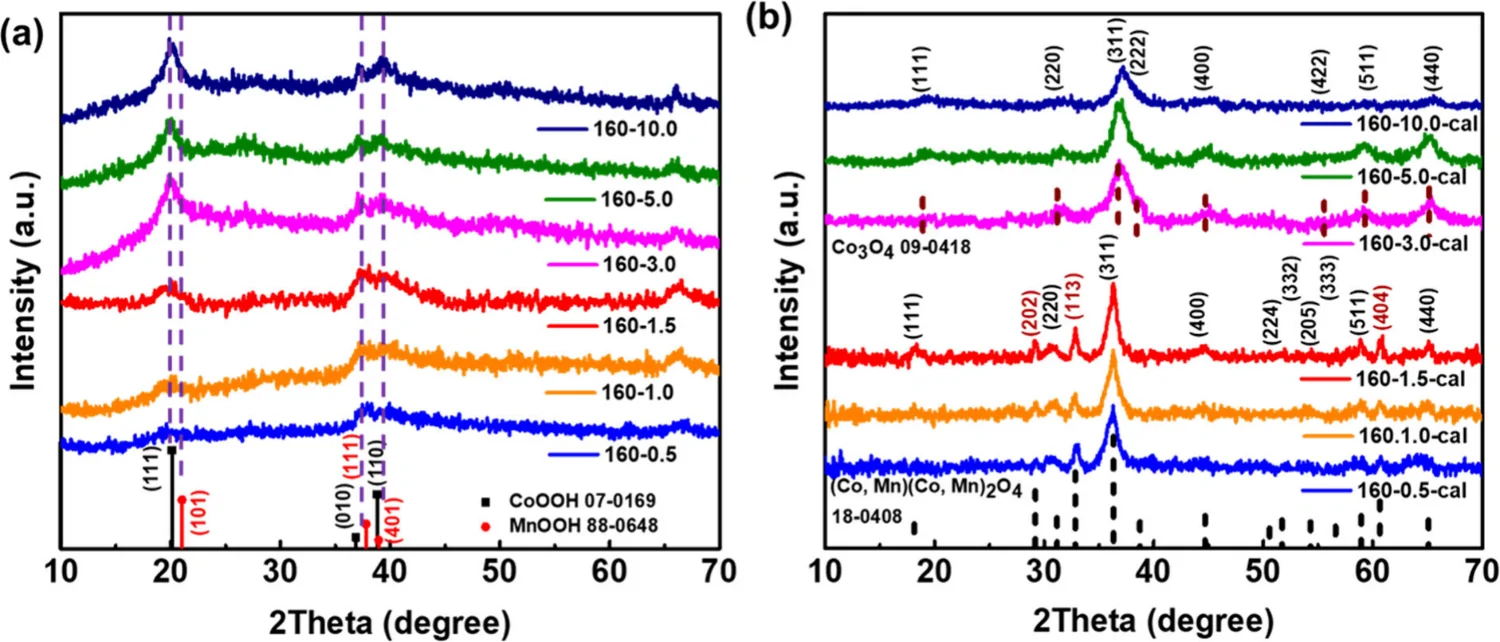
Powder X-ray diffraction (PXRD) patterns of LRS system products synthesized at 160 °C with varying water additions: (a) as-prepared (uncalcined) and (b) after calcination at 500 °C. Dotted lines indicate peak positions for the phases assigned using the corresponding JCPDS reference patterns (listed in the figure).

For the uncalcined products (Figure 2a), all samples (160–0.5 to 160–10.0) exhibit weak, broadened reflections, consistent with predominantly amorphous or nanocrystalline oxyhydroxide intermediates. Broad features at 2θ ≈ 18–20° and 36–38° can be assigned to CoOOH (JCPDS 07-1069) and MnOOH (JCPDS 88-0648), in line with the oxyhydroxide phases typically obtained from ARP.^25^ Because the uncalcined products are poorly crystalline, definitive identification of the final spinel phase is not straightforward at this stage; therefore, we analyze the calcined samples to determine the resulting structures clearly.

After calcination (Figure 2b), low-water samples produce the metastable tetragonal spinel. Specifically, 160–0.5-cal, 160–1.0-cal, and 160–1.5-cal exhibit the characteristic tetragonal reflections (111), (202), (220), (113), (311), (400), (511), (404), and (440), matching (Co,Mn)­(Co,Mn)_2_O_4_ (JCPDS 18-0408).^29^ In contrast, increasing water content triggers a clear tetragonal-to-cubic transition. Beginning at 160–3.0-cal, the (311) reflection shifts from 2θ = 36.402° (tetragonal) to 2θ = 36.846° (cubic), while tetragonal-only peaks (202), (113), and (404) vanish, consistent with formation of the thermodynamic cubic spinel Co_3_O_4_ (JCPDS 09-0418).^26^ The 160–5.0-cal and 160–10.0-cal samples likewise index to cubic Co_3_O_4_. Notably, peak broadening is observed at higher water loadings, suggesting smaller crystallites and/or increased structural disorder.^30^ The emergence of cubic Co_3_O_4_ under 3–10 mL water is consistent with conventional aqueous ARP behavior,^26^ supporting the central design principle that minimizing liquid volume is required to prevent re-equilibration of the structure, selectively producing the tetragonal phase. Accordingly, selective access to tetragonal CMO requires a low liquid-to-solid ratio, η ≤ 0.81 μL mg^–1^ (≤1.5 mL) at 160 °C.

The structural trends above indicate that increasing the initial water addition switches the phase selectivity from the metastable tetragonal spinel to the thermodynamically stable cubic spinel. We hypothesize that complete removal of water during heating is also important for phase selectivity since the heating reaction is not sealed. To investigate the effect of water dehydration during reaction on phase evolution, four different dehydration rates were studied: fast dehydration (crucible without lid), moderate dehydration (crucible covered with a lid), slow dehydration (sealed crucible), and near-zero dehydration (Teflon autoclave providing a quasi-sealed environment). Note that other samples mentioned in this study follow the moderate dehydration condition. The quantitative comparison of the different dehydration rates and the residual water contents associated with each dehydration condition is mentioned in Figure S2 and Table S2.

As shown in Figure 3, under fast and moderate dehydration conditions when water is completely removed, the XRD pattern exhibits tetragonal features, including the characteristic diffractions at (202), (220), and (113), as well as a (311) peak centered at 2θ = 36.40°, consistent with tetragonal CMO. Further, as more water remains in the system, for slow and near-zero samples, the tetragonal characteristic peaks of (202) and (113) are weakened and disappear, in exchange for the broad (220) characteristic of cubic spinel (Figure 3a). The (311) peak shifts to a higher angle, reaching 2θ = 36.66° for the near-zero dehydration sample (Figure 3b). This shift toward the cubic peak position indicates progressive loss of tetragonal distortion and structural relaxation toward the thermodynamically stable cubic spinel. These results suggest that complete removal of water is equally necessary to kinetically trap Mn^3+^ by limiting ion transport and redox re-equilibration. Therefore, tetragonal phase stabilization in the LRS system is governed not only by the initial water addition but also by water removal during heating.

**Figure 3:**
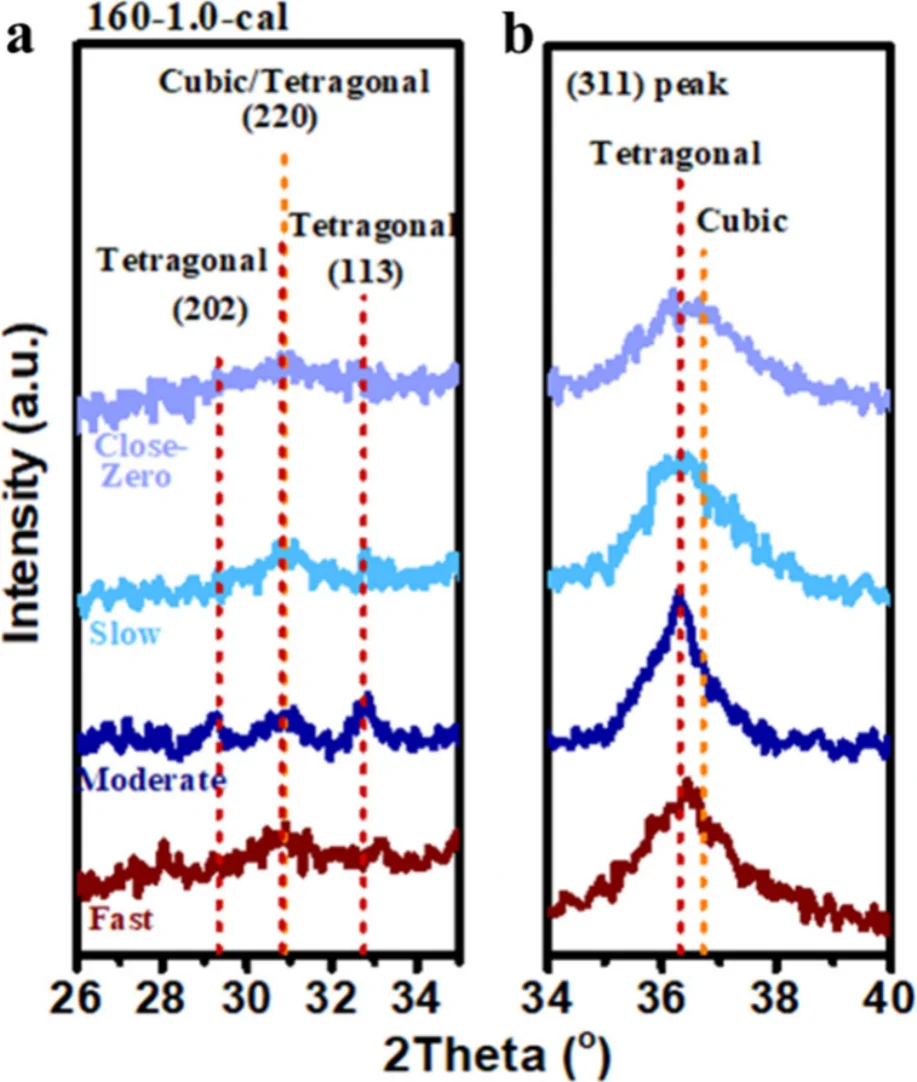
Effect of different water-removal rates on phase evolution of 160–1.0-cal in the LRS system, (a) is the characteristic diffractions at (202), (220), and (113) for tetragonal spinel. And (b) is the (311) peak shift from tetragonal to cubic spinel.

To further evaluate the influence of bulk composition on phase selection, the Co ratios of representative samples were determined by Inductively Coupled Plasma (ICP) analysis. In the Co–Mn spinel system, phase stability is known to depend on both composition and cation valence states.^31,32^ Accordingly, determining the Co ratio is important for distinguishing whether the observed tetragonal phase originates from compositional variation or from differences in oxidation state and local structure. The measured Co:Mn molar ratios are summarized in Table S3. The results show that all samples remain cobalt-rich, with Co:Mn ratios ranging from 1.91:1 to 2.90:1 for the low liquid-to-solid ratio samples (160–0.5-cal, 160–1.0-cal, and 160–1.5-cal) exhibiting spinel composition close to Co_2_MnO_4_.

In particular, Co_2_MnO_4_ is typically stabilized in the cubic spinel structure under equilibrium conditions, whereas the tetragonal phase is hard to stabilize. The formation of tetragonal spinel under the same cobalt-to-manganese composition needs more complex and specific redox-controlled equilibrium in the synthesis.^33^ Further, the samples synthesized with larger liquid-to-solid ratios, η ≥ 1.61 μL mg^–1^ (≥3 mL), exhibit progressively higher Co:Mn ratios (2.3–2.9:1). These results are similar to those typically observed for conventional aqueous ARP products (Co:Mn ≈ 3:1).^26^ Therefore, based on the resulting composition, a cubic spinel phase would be expected.

We next quantify how water loading impacts conversion by calculating product yields (mass production yields), which is based on the isolated product mass relative to the theoretical recovery based on the redox stoichiometry (details in Section S2). The oxidant, KMnO_4_, was treated as the limiting precursor. Assuming the stoichiometry of Co_2_MnO_4_ spinel is used, which is consistent with the ICP-derived Co:Mn ratio of 2.17 ± 0.10 (for the 160–1.0 sample, Table S3), changing the water removal rate with the same initial water does not produce much variation in the product yield (average of 82.6 ± 4.1%, Table S4), which might be due to the similar precursor concentration and reaction rate. Under a low liquid-to-solid ratio environment, which selectively produces tetragonal CMO, the yields are high: 80.3 ± 2.7%, 86.3 ± 3.0%, and 71.4 ± 2.4% for 160–0.5, 160–1.0, and 160–1.5, respectively. Notably, the optimized LRS condition (160–1.0) delivers the highest yield (86.3 ± 3.0%), which is ∼27× higher than that of conventional aqueous ARP (3.2 ± 1.8%). In contrast, increasing the water volume to 3–10 mL decreases yield, reaching 15.6 ± 3.6% at 160–10.0.

Transmission electron microscopy (TEM) was used to compare representative tetragonal and cubic products (Figure 4). For the tetragonal sample 160–1.5-cal, TEM reveals aggregates of nanosized crystallites with irregular morphology (Figure 4a). The corresponding selected-area electron diffraction (SAED) pattern acquired along the [032] zone axis (Figure 4b) displays reflections assignable to the (200) and (123) planes, consistent with the lower-symmetry tetragonal spinel lattice (*I*4_1_/*amd*) and in agreement with XRD. High-resolution TEM (HRTEM) further shows lattice fringes with an interplanar spacing of ≈3.97 Å, matching the (200) plane (Figure 4c), confirming that the local crystallography is consistent with bulk tetragonal CMO.

**Figure 4:**
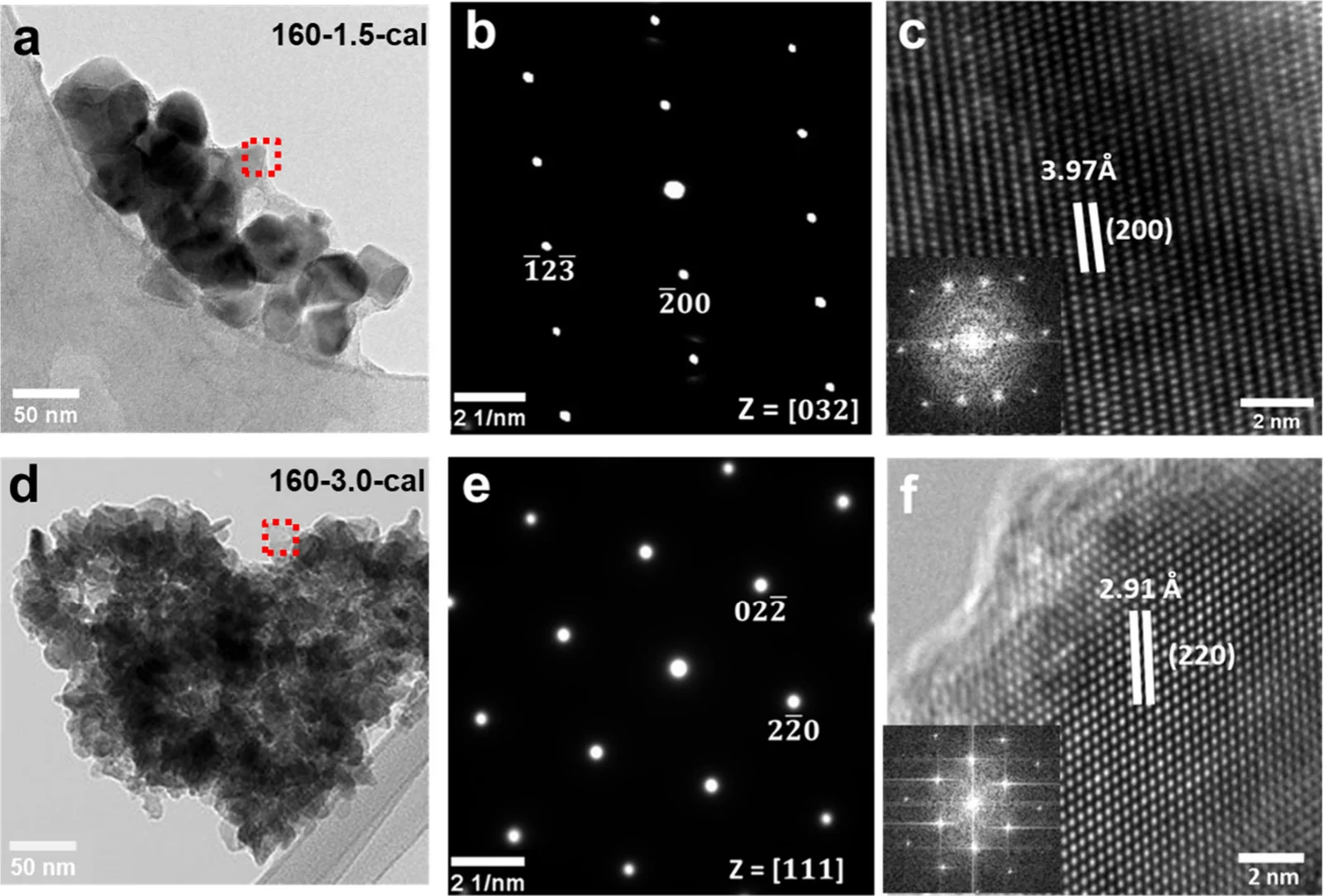
TEM, SAED, and HRTEM characterization of representative LRS system products. (a–c) 160–1.5-cal (tetragonal spinel): (a) TEM image, (b) SAED pattern acquired from the region marked in (a), and (c) HRTEM image showing the measured lattice spacing. (d–f) 160–3.0-cal (cubic spinel): (d) TEM image, (e) SAED pattern acquired from the region marked in (d), and (f) HRTEM image showing the measured lattice spacing. Measured *d*-spacings are indicated in (c) and (f).

In contrast, the cubic sample 160–3.0-cal consists of aggregated, irregular particles (Figure 4d). Its SAED pattern (Figure 4e), collected along the [111] zone axis, can be indexed to the (220) and (022) planes characteristic of the cubic spinel structure (*Fd*-3 *m*). Consistently, HRTEM resolves lattice fringes with *d* ≈ 2.90 Å, corresponding to the (220) plane (Figure 4f). Together, these analyses corroborate the XRD results and verify that a low liquid-to-solid ratio, η ≤ 0.81 μL mg^–1^ (≤1.5 mL), yields tetragonal CMO, whereas higher water loading (≥3.0 mL) produces the cubic spinel phase. Previous studies have reported that Mn-rich Co–Mn spinels preferentially adopt the tetragonal *I*41*/amd* structure, whereas Co-rich compositions tend to stabilize the cubic *Fd*3̅*m* phase.^34^ While the present ICP results show a similar compositional trend (Table S3), the formation of a tetragonal phase in the present study cannot be attributed solely to compositional effects, as compositions with Co_2_MnO_4_-like stoichiometry are commonly reported to crystallize in the cubic spinel structure.

### 2.3 X-ray Absorption Spectroscopy for Tetragonal CMO

To clarify the origin of the metastable tetragonal spinel, we employed X-ray absorption spectroscopy (XAS) to quantify the oxidation states and local coordination environments of Co and Mn (Figure 5). Both XANES and EXAFS were analyzed to track how the initial water addition affects the redox outcome of the LRS system.

**Figure 5:**
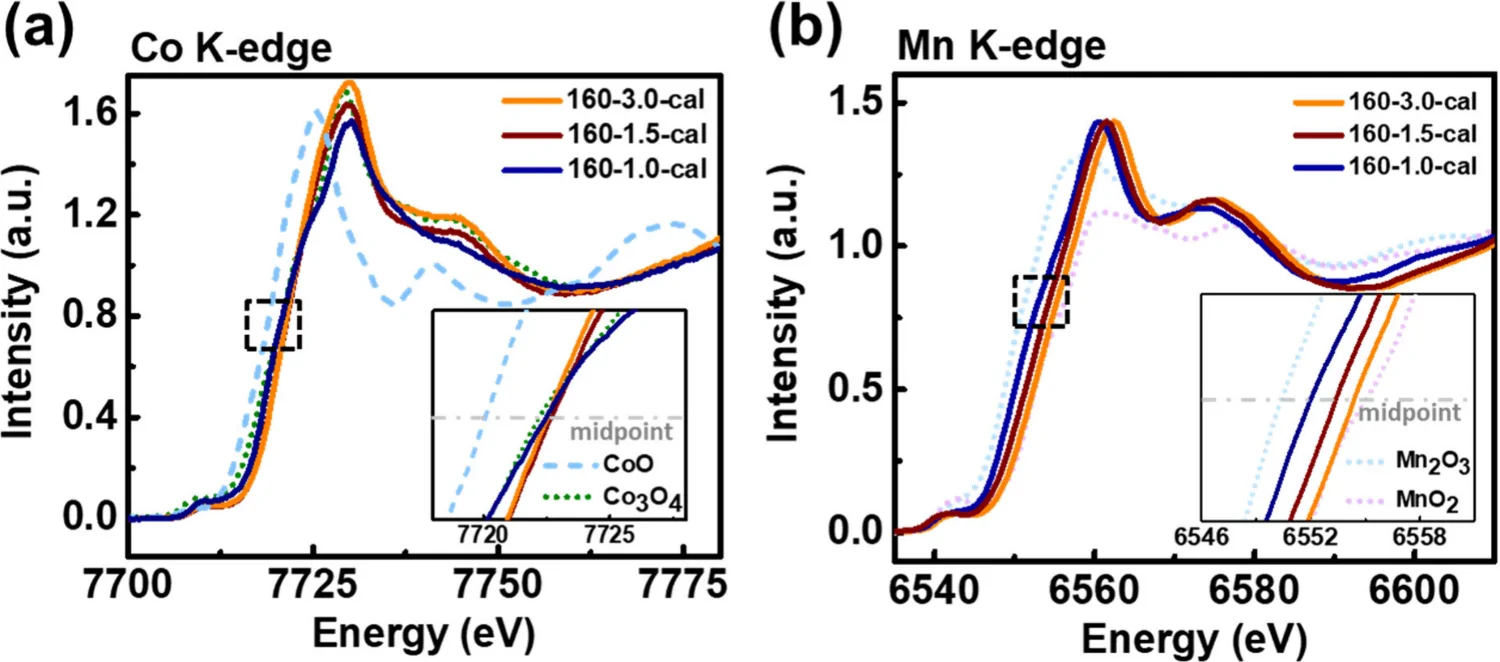
Co and Mn K-edge X-ray absorption spectroscopy (XAS) of representative LRS system products (160–1.0-cal, 160–1.5-cal, 160–3.0-cal) with reference standards. (a) Normalized Co K-edge XANES spectra compared with CoO and Co_3_O_4_. (b) Normalized Mn K-edge XANES spectra compared with Mn_2_O_3_ and MnO_2_.

In the Co K-edge XANES (Figure 5a), the low-water sample 160–1.0-cal exhibits a half-height (midpoint) edge at ∼7720 eV (inset, Figure 5a), matching the spectral signature of Co_3_O_4_ and corresponding to an average Co oxidation state of +2.67, indicative of a mixed-valence Co^2+^/Co^3+^ population. With increasing water addition, the Co edge shifts to higher energy: ∼7721.45 eV (+2.8) for 160–1.5-cal and ∼7721.70 eV (+2.9) for 160–3.0-cal (inset, Figure 5a). These values were obtained by interpolating the edge positions between CoO (Co^2+^) and Co_3_O_4_ (Co^2.67+^) (Figure S3a).^35^

An analogous trend is observed for Mn K-edge XANES (Figure 5b). The midpoint edge appears at ∼6551.8 eV for 160–1.0-cal and shifts to ∼6553.2 eV for 160–1.5-cal and ∼6554.2 eV for 160–3.0-cal (inset, Figure 5b). Using a linear calibration between Mn_2_O_3_ (Mn^3+^) and MnO_2_ (Mn^4+^) based on the half-height method (Figure S3b),^15^ the average Mn oxidation states are estimated as ∼+3.3 (160–1.0-cal), ∼+3.6 (160–1.5-cal), and ∼+3.9 (160–3.0-cal), corresponding to the Mn^3+^/Mn^4+^ ratios 7/3, 4/6, and 1/9, respectively. We acknowledge that a minor Mn^2+^ contribution may be present, in relatively small portion. Therefore, the reported Mn^3+^/Mn^4+^ ratios should be used as representations derived from the average Mn valence estimated by XANES. We further note that variations of Mn oxidation states might induce local structural distortions or defect formation while preserving the overall oxide framework.^36^ Collectively, these results suggest that a low liquid-to-solid ratio favors Mn^3+^-rich character, whereas a higher liquid-to-solid ratio promotes Mn^4+^-rich character.

To further elucidate how the water environment modulates the local Mn coordination associated with tetragonal versus cubic spinel formation, we analyzed the Mn K-edge *k* ^3^-weighted FT-EXAFS spectra (Figure 6a). The first-shell Mn–O feature (apparent *R* ≈ 1.5 Å) for 160–1.0-cal is broader and less intense than those of the higher-water samples, indicating a distribution of Mn–O bond lengths. This behavior is consistent with a distorted MnO_6_ octahedral environment, i.e., Jahn–Teller-active Mn­(III) in a high-spin d^4^ configuration (t_2_g^3^ eg^1^), which induces anisotropic Mn–O bonding.^37,38^ As the water content increases to 1.5 and 3.0 mL, the Mn–O peak becomes narrower and shifts slightly to shorter apparent *R* (from ∼1.5 Å in 160–1.0-cal to ∼1.41 Å in 160–3.0-cal), consistent with the higher average Mn oxidation state inferred from XANES (Figure 5b; ∼+3.3 → ∼+3.9), which generally shortens Mn–O bonds.

**Figure 6:**
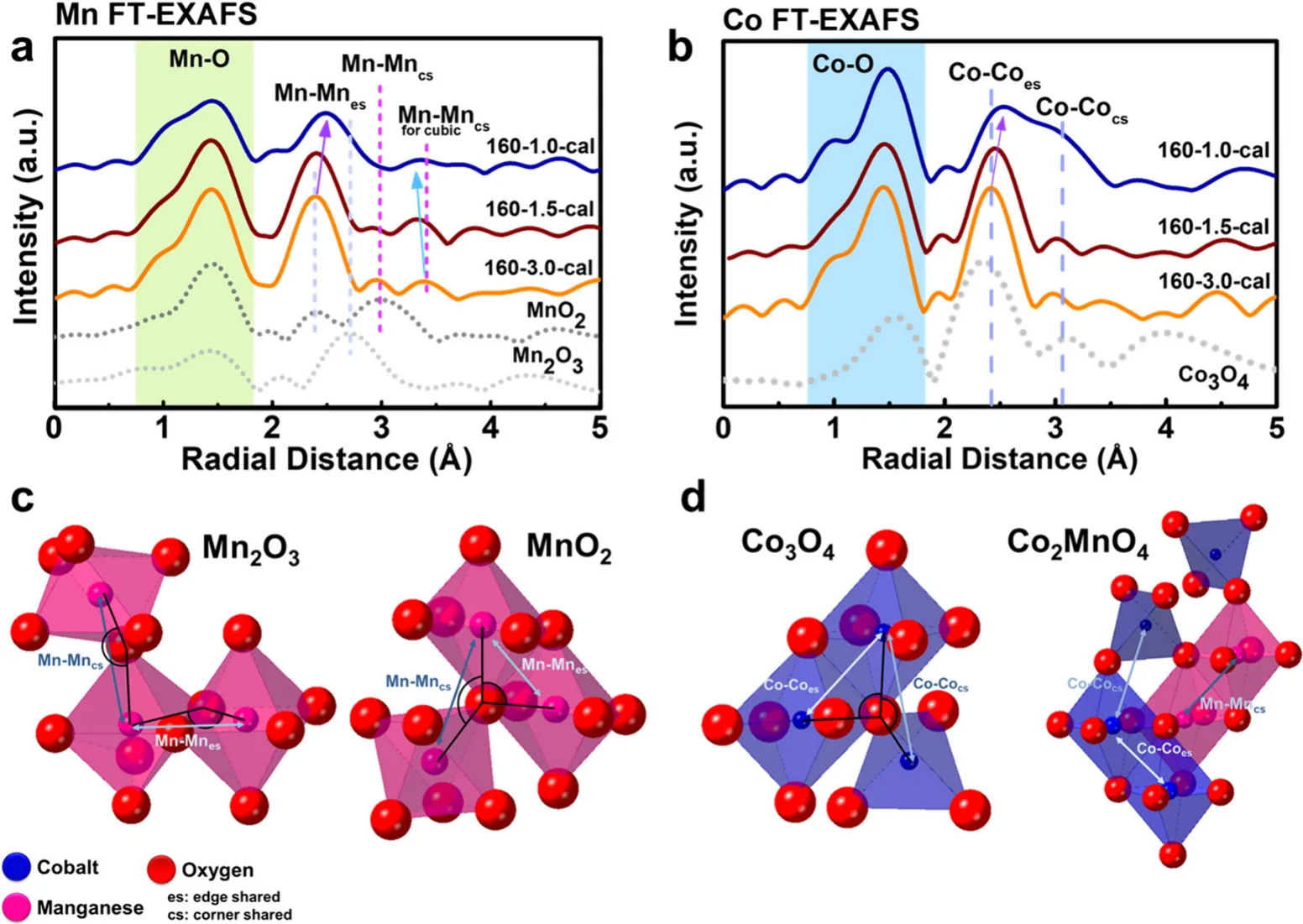
*k* ^3^-weighted Fourier-transformed EXAFS (FT-EXAFS) spectra of (a) Mn K-edge and (b) Co K-edge for representative CMO samples (160–1.0, 160–1.5, 160–3.0) compared with reference compounds (MnO_2_ and Mn_2_O_3_ for Mn; Co_3_O_4_ for Co). (c, d) Schematic illustrations highlighting characteristic metal–metal distances and corresponding edge-sharing (≈2.4–2.7 Å) and corner-sharing (≈3.1 Å) contributions in Mn–Mn and Co–Co coordination environments.

In the second-shell region, the edge-sharing Mn–Mn contribution (Mn–Mn_edge shared_, apparent *R* ≈ 2.5–2.8 Å) for 160–1.0-cal is also broader and shifted to larger apparent *R* (from ∼2.36 Å in 160–3.0-cal to ∼2.51 Å in 160–1.0-cal). Comparison with reference spectra (Figure 6a) and crystallographic models of MnO_6_ (Figure 6c) indicates that Mn­(III)-rich motifs can exhibit larger Mn–Mn_edge shared_ distances than Mn­(IV)-dominated frameworks. In addition, the corner-sharing Mn–Mn feature, typically evident in MnO_2_ and related Mn­(IV) oxides,^39^ is strongly attenuated here, suggesting reduced corner-sharing connectivity for Mn in the low-water sample. Collectively, these EXAFS signatures support the conclusion that 160–1.0-cal contains a higher fraction of Jahn–Teller-distorted Mn­(III)­O_6_ units, which contribute to the tetragonal spinel phase forming under limited initial water addition.

The Co K-edge *k* ^3^-weighted FT-EXAFS spectra (Figure 6b) further show that water loading alters the Co local structure, most noticeably beyond the first shell. The first-shell Co–O feature (apparent *R* ≈ 1.5–1.7 Å) is similar for 160–1.0-cal, 160–1.5-cal, and 160–3.0-cal, indicating comparable primary Co–O coordination. In contrast, the second-shell region displays two Co–Co contributions near apparent *R* ≈ 2.5 Å (Co–Co_edge shared_) and 3.0 Å (Co–Co_corner shared_), consistent with spinel-type Co–Co correlations (Co_3_O_4_ reference; Figure 6d).^40^ Importantly, the Co–Co_edge shared_ peak systematically broadens and shifts to a larger apparent distance as water decreases (∼2.39 Å for 160–3.0-cal, ∼2.42 Å for 160–1.5-cal, and ∼ 2.51 Å for 160–1.0-cal), paralleling the Mn–Mn_edge shared_ trend and supporting coupled distortion between the CoO_6_ and MnO_6_ sublattices under low-water conditions. Considering Co:Mn ≈ 2.2:1 (based on ICP elemental analysis), together with the XANES and FT-EXAFS results, the local structure is consistent with a Co_2_MnO_4_-like spinel framework (Figure 6d). Based on literature reports for Co–Mn spinels, Mn is expected to predominantly occupy octahedral sites, while Co can occupy both tetrahedral and octahedral sites.^41,42^ The schematic shown in Figure 6d is therefore intended as a structural model consistent with the experimental observations rather than a conclusive determination of cation site occupancy.

### 2.4 Selective Formation of Mn^3+^ and the Tetragonal Spinel Phase

In conventional aqueous ARP, permanganate reduction typically converges toward Mn­(IV)-rich products, consistent with formation of the thermodynamically favored cubic spinel (see eq 1).^26^

1 $$9{{\mathrm{Co}}^{2+}}_{\left(\mathrm{aq}\right)}+3{{{\mathrm{MnO}}_{4}}^{-}}_{\left(\mathrm{aq}\right)}+14H_{2}O_{\left(l\right)}\to {\mathrm{Co}}_{9}{\mathrm{Mn}}_{3}O_{26}H_{13\left(s\right)}+15{H^{+}}_{\left(\mathrm{aq}\right)}$$

In contrast, the LRS method yields a substantial Mn­(III)/Mn­(IV) population by XAS under low-water conditions, which is crucial because Jahn–Teller-active Mn­(III) in octahedral sites provides a direct structural driving force for the tetragonal spinel. Since the low liquid-to-solid samples are still cobalt-rich, the emergence of the tetragonal phase cannot be attributed solely to compositional differences. Combined with the Mn K-edge XANES results, the formation of tetragonal spinel in cobalt-rich, Co_2_MnO_4_-like stoichiometry suggests that Mn^3+^ incorporation and the associated Jahn–Teller distortion are the primary factors governing tetragonal phase formation in the present LRS process.

This selectivity cannot be attributed simply to permanganate concentration: when comparing 1 mL vs 3 mL water additions, the MnO_4_^–^ concentration remains saturated in both cases, and thus cannot explain the divergent Mn oxidation outcomes. Instead, the key difference introduced by trace water is a more acidic local environment. Using a pH indicator with oversaturated CoSO_4_ in 1 mL of DI water, we estimate pH ≈ 2–3, whereas conventional ARP under larger water volumes is typically pH ≈ 5–6. For comparison, an oversaturated KMnO_4_ solution shows a much weaker ability to shift pH, indicating that the acidification is dominated by the concentrated CoSO_4_-containing medium rather than permanganate itself.

The pH-dependent Nernst analysis summarized in Table 1 captures how this acidity shift biases Mn redox chemistry (see the details in SI, Section S2): the calculated *E*°(Mn­(VII)/Mn­(IV)) and *E*°(Mn­(VII)/Mn­(III)) evolve with pH such that Mn­(III) becomes increasingly competitive during MnO_4_^–^ reduction. At the same time, the Mn­(IV)/Mn­(III) relation further tilts toward Mn­(III) under more acidic conditions (the sign change in Table 1 indicates that Mn­(IV) → Mn­(III) becomes favored at lower pH).

**Table 1:** pH-Dependent Nernst Reduction Potentials of Mn­(VII)/Mn­(IV), Mn­(VII)/Mn­(III), and Mn­(IV)/Mn­(III) Redox Couples^a^

pH Value	*E*° Mn(VII)/Mn(IV)	*E*° Mn(VII)/Mn(III)	Δ*E*°^b^	*E*° Mn(IV)/Mn(III)
6	1.22 V	0.795 V	0.424 V	–0.470 V
5	1.30 V	0.913 V	0.385 V	–0.233 V
4	1.38 V	1.032 V	0.345 V	0.004 V
3	1.46 V	1.150 V	0.306 V	0.240 V
2	1.53 V	1.268 V	0.266 V	0.477 V

a This equation only concerns pH, with the assumption of [MnO_4_^–^] = saturated concentration; [Mn^3+^]_aq_ ≈ 0.

b Δ*E*° = *E*° Mn­(VII)/Mn­(IV) – *E*° Mn­(VII)/Mn­(III).

However, the favorability alone does not guarantee the incorporation and stabilization of Mn­(III), as Mn­(III) species readily undergo disproportionation in aqueous environments. First, a low liquid-to-solid ratio (η) generates a highly concentrated and acidic environment, which favors permanganate reduction toward Mn­(III)-containing intermediates. Furthermore, water dehydration suppresses solution-mediated re-equilibration, thereby facilitating the incorporation of Mn­(III) into the oxide framework. Collectively, these findings indicate that controlling the reaction environment through limited water availability and further dehydration enables the stabilization of Mn­(III), thereby directing the selective formation of the metastable tetragonal spinel phase.

## 3 Electrochemistry Application for OER Catalyst

Electrochemical measurements in 1.0 M KOH were conducted to examine how spinel symmetry and lattice distortion impact alkaline OER activity. As summarized in Figure S4, the tetragonal 160–1.0 sample consistently outperforms the cubic 160–3.0 analog, showing lower overpotentials and faster kinetics both before and after calcination (Figure S4a). At 10 mA cm^–2^, 160–1.0 requires an overpotential of 460 mV, compared with 498 mV for 160–3.0; the same trend also occurs for the calcined samples. The Tafel slope for 160–1.0 (72.8 mV dec^–1^) is markedly smaller than that of 160–3.0 (113.8 mV dec^–1^) (Figure S4b), indicating more favorable OER kinetics. The electrochemical impedance spectroscopy (EIS) further supports improved interfacial charge transfer for 160–1.0, which exhibits a smaller semicircle and a reduced *R*_ct_ ≈ 11.3 Ω, relative to 160–3.0 (≈ 14.2 Ω) and blank FTO (≈ 19 Ω) (Figure S4c). In parallel, the electrochemical surface area (ECSA) estimated from *C*_dl_ (Figure S4d; Table S5) is nearly 2-fold higher for 160–1.0, consistent with a larger population of accessible surface sites.

Importantly, the performance enhancement is most reasonably linked to the structural contrast between cubic and tetragonal spinels. The tetragonal phase is less symmetric and originates from lattice distortion, which is expected to introduce a higher density of nonequivalent metal–oxygen environments, including defects and undercoordinated sites, relative to the more symmetric cubic framework.^33,43^ Such structural heterogeneity can increase the number and diversity of catalytically competent surface motifs and can also facilitate charge transfer across the oxide/electrolyte interface, consistent with the lower *R*_ct_ and improved kinetics observed for 160–1.0. Together, these trends support that the tetragonal distortion itself—rather than solely differences in composition—contributes to the enhanced alkaline OER activity.

## 4 Conclusions

Metastable phases of multinary metal oxides are difficult to access selectively and in high yield. Here, we demonstrate that a liquid-assisted redox synthesis (LRS) strategy enables controlled formation of metastable tetragonal Co–Mn spinel (CMO) through a combination of low liquid-to-solid ratio, η ≤ 0.81 μL mg^–1^ (≤1.5 mL), and complete dehydration. A schematic of the initial water addition establishes a clear phase map: ≤1.5 mL selectively yields the metastable tetragonal CMO spinel, whereas ≥3.0 mL produces the thermodynamically stable cubic spinel, with an optimized yield of 88.0% (vs 3.2% for conventional aqueous ARP). Mechanistically, the low liquid-to-solid ratio (η = 0.27–0.81 μL mg^–1^) creates a minimally hydrated, highly concentrated, and more acidic microenvironment that biases permanganate reduction toward a mixed Mn­(III)/Mn­(IV) population rather than Mn­(IV)-only outcomes typical of bulk aqueous processing. Furthermore, the complete dehydration suppresses the Mn­(III)/Mn­(IV) re-equilibration and enables trapping of Mn­(III), which is essential for stabilizing the metastable tetragonal structure. XAS further confirms Mn­(III) enrichment and Jahn–Teller-distorted MnO_6_ motifs, providing a structural basis for stabilizing the tetragonal lattice and coupled changes in Co coordination. Finally, the tetragonal phase exhibits improved alkaline OER activity relative to the cubic analog, highlighting the functional value of kinetically accessed distortion.

## 5 Experimental Section

### Synthesis of CMO Using the LRS System Method

The precursor powders were prepared by separately grinding 5.5 mmol of CoSO_4_·7H_2_O (Showa) and 2 mmol of KMnO_4_ (J.T. Baker) using a mortar and pestle. After obtaining fine powders, the two precursors were combined and ground together for 5–10 min, followed by the dropwise addition of deionized water (DI water) (18.2 MΩ·cm) to the powder mixture. After mixing with DI water for 5–10 min, the resulting slurry was transferred to a crucible and subjected to heat treatment at 90, 120, 160, and 200 °C for 2 h. The dark brown powders obtained were washed with DI water to remove unreacted precursors, and the product yield (%) was determined (detailed calculation in SI, Section S1). Controlled amounts of DI water (0.5, 1.0, 1.5, 3.0, 5.0, and 10.0 mL) were added to adjust the liquid-to-solid ratio during synthesis. The samples are denoted as T-x, where T represents the synthesis temperature (in °C) and x denotes the amount of water added (in mL). For instance, a sample synthesized at 160 °C with 1.0 mL of water is labeled as 160–1.0. Further, all calcined samples (at 500 °C for 2 h) are denoted as T-x-cal.

To quantitatively describe the amount of water added during synthesis, the initial liquid-to-solid ratio (η) was calculated following previous reports on liquid-assisted synthesis.^22^ The η is defined as

2 $$\eta =\frac{V_{water}\left(\mu L\right)}{m_{totalprecursor}\left(mg\right)}$$

The total precursor mass of CoSO_4_·7H_2_O (5.5 mmol) and KMnO_4_ (2.0 mmol) used in this study was 1.862 g (1862 mg). The calculated η values corresponding to different amounts of water addition are summarized in Table S1.

Because the system is not sealed, the water removal rate was adjusted by varying the sealing condition of the reaction vessel during heating (open, partially sealed, and sealed configurations), thereby controlling the amount of water remaining in the system. The sample used for the study is 160–1.0. The variation code mentioned is

- Crucible without lid → fast (fd)
- Crucible with lid → moderate (md)
- Sealed crucible → slow (sd)
- Teflon autoclave or quasi-sealed → near-zero (cd)

To quantitatively characterize the dehydration environments associated with these conditions, additional gravimetric measurements were conducted using a blank system containing only 1 mL of deionized water in the same porcelain crucible geometry employed for the synthesis (diameter, 4.5 cm; height, 4.0 cm). The resulting comparison data of the dehydration rates under the mentioned dehydration conditions are presented in Figure S2. Furthermore, the residual water remaining after 2 h of heating for different dehydration conditions was experimentally determined and is summarized in Table S2. These measurements provide a practical quantitative basis for distinguishing the different dehydration environments and facilitate reproducibility across the laboratories.

### Characterization

X-ray diffraction (XRD) patterns were recorded using a Bruker D2 Phaser X-ray diffractometer with a Cu–Kα light source (λ = 1.5406 Å). Transmission electron microscopy (TEM) was conducted with a Tecnai F20 G2MAT S-TWIN field emission gun transmission electron microscope at 200 kV. The elemental analysis was carried out with an inductively coupled plasma mass spectrometer (ICP-MS) using a THERMO-ELEMENT XR spectrometer. Samples were dissolved in a solution composed of a volume ratio of DI water:HNO_3_:H_2_O_2_ = 3:2:1, respectively. The Synchrotron XAS measurement was done at Synchrotron Light Research Institute (SLRI), Thailand, under beamline BL5.2: SUT-NANOTEC-SLRI XAS beamline (XAS).

### Electrochemical Tests

Electrochemical tests were conducted using an Autolab PGSTAT302N with a Pt plate (for counter) and a Hg/HgO reference electrode. The catalyst was coated onto a fluorine-doped tin oxide (FTO) conductive glass substrate, which was used as the working electrode. The working electrode was prepared by dispersing 10 mg of the electrocatalyst in 5 mL of a deionized water/isopropanol mixture (v/v = 3:1) containing 10% PiperION anion exchange ionomer (Anion Exchange Dispersion, Fuel Cell), followed by sonication for 30 min to obtain a homogeneous ink. Subsequently, 100 μL (applied in two steps of 50 μL) of the ink was drop-cast onto an FTO substrate (0.5 × 0.5 cm^2^) to cover the top surface of the electrodes. The oxygen evolution reaction (OER) activities were assessed using linear sweep voltammetry (LSV) at a scan rate of 5 mV s^–1^. The potentials measured against the potential of Hg/HgO were converted to the reversible hydrogen electrode (RHE) according to the reference electrode calibration provided below (eq 3):^26^

$$E_{\mathrm{RHE}}=E_{\mathrm{Hg}/\mathrm{HgO}}+0.098+0.059\times \mathrm{pH}$$ 3

All electrochemical measurements were conducted in a 1 M KOH electrolyte with *iR*-correction. The *iR* correction of the electrode potential was performed using the cell ohmic drop (*R*) obtained from electrochemical impedance spectroscopy (EIS), shown in eq 4. EIS was recorded by applying a 5 mA amplitude in the frequency range from 100 kHz (high frequency) to 10 Hz (low frequency).

4 $$E_{iR\mathrm{‐corrected}}=E_{\mathrm{RHE}}-iR$$

*E*_RHE_ and *E*_*iR*‑corrected_ are the measured and corrected potentials in V, respectively, while *i* is the measured current in A, and *R* is the electrolyte resistance in Ω.

Tafel slopes were obtained by plotting the overpotential of each sample against the logarithm of the current density, log *i* (mA cm^–2^). The electrochemically active surface area (ECSA) was determined using cyclic voltammetry (CV) within the potential range corresponding to the electric double layer. Measurements were performed on the electrode at varying scan rates (30, 50, 70, and 100 mV s^–1^) over a potential of 0.955–1.055 V versus RHE in 1 M KOH. By plotting the current density difference at 1.005 V against the scan rates, the slope corresponds to *C*_dl_ for each sample. The specific capacitance value of a flat surface (*C*_s_) was estimated from the fluorine-doped tin oxide (FTO) blank substrate. The ECSA was calculated by the equation as follows (eq 5):

5 $$ECSA=\frac{C_{dl}}{C_{s}}$$

The resulting *C*_dl_ and ECSA values for all samples are summarized in Table S5.

## Supplementary Material



## Data Availability

The data supporting this article have been included as part of the Supporting Information.

## Notes

The calculation of reduction potential (*E*°) using the Nernst equation; % yield calculation of the product; Co:Mn composition ratios determined by ICP analysis; XRD pattern for the sample synthesized under 160 °C without water addition (160–0); corresponding oxidation number determined by linear fitting of the absorption-edge positions in XANES spectra; electrochemical performance of the LRS system samples (PDF)
