# Supplementary material for: Liquid-Assisted Redox Synthesis Enables High-Yield Metastable Tetragonal Spinel Oxides
Source: ACS Mater Au. 2026 Jul 22;6(5):1162–71. doi: 10.1021/acsmaterialsau.6c00107 (PMC13564152; doi:10.1021/acsmaterialsau.6c00107)
Supplement: Supplementary file 1 [file mg6c00107_si_001.pdf]

Supporting Information

Liquid-assisted Redox Synthesis Enables High-yield Metastable Tetragonal Spinel Oxides

Yanita Devi[a] and Chun-Hu Chen[a,b,*]

[a] *Department of Chemistry, National Sun Yat-sen University, Kaohsiung, Taiwan 80424*

[b] *Green Hydrogen Research Center, National Sun Yat-sen University, Kaohsiung, Taiwan 80424*

* *Corresponding author. E-mail address: chunhu.chen@mail.nsysu.edu.tw*

S1. Calculation of the percentage yield

The product yield of cobalt–manganese oxide (CMO) was determined from the isolated mass of the dried product. Each batch was prepared from $CoSO_4 \cdot 7H_2O$ and $KMnO_4$ with a total of 5.5 mmol Co and 2.0 mmol Mn (combined precursor mass = 1.862 g). Assuming the spinel composition Co_2MnO_4, which requires a Co:Mn molar ratio of 2:1, the initial precursor feed ratio (Co:Mn = 2.75:1) indicates that $CoSO_4 \cdot 7H_2O$ was present in excess, while $KMnO_4$ was treated as the stoichiometrically limiting precursor. The crystal water associated with $CoSO_4 \cdot 7H_2O$ and the soluble counter ions (K^+ and SO_4^{2-}) were excluded from the yield calculation because they are removed during the washing process and do not contribute to the final oxide product.

After subtracting the mass contributions of crystal water and soluble counter ions, the remaining precursor weight is 0.563 g. Because the LRS process involves redox precipitation, dehydration, and the removal of soluble by-products, the yield values reported in this work were defined as mass production yields based on the recoverable oxide-forming precursor mass rather than conventional stoichiometric yields assuming complete conversion to a single ideal product composition. The product yield was therefore defined as follows (**Eq. S1**):

$$\text{Mass production yield} = \frac{The\ resulting\ CMO\ from\ LAG{-}ARP\ (gram)}{The\ remining\ precursor\ mass\ (gram)} \quad \textbf{Eq. S1}$$

$$= \frac{The\ resulting\ CMO\ from\ LAG{-}ARP\ (gram)}{0.563\ gram}$$

The mass product (g) and the percent yield (%) are shown in **Table S4**.

S2. The Nernst-calculated standard reduction potentials for the Mn(VII)/Mn(IV), Mn(VII)/Mn(III), and Mn(IV)/Mn(III) redox couples

The standard potential for the reduction of Mn^{7+} to stable Mn^{4+} in the solution at 25°C is shown below:[3]

$$MnO^{-}_{4\,(aq)} + 4H^{+} + 3e^{-} \leftrightarrow MnO_{2\,(s)} + 2H_2O \qquad E^o = 1.70\ V \qquad \textbf{Eq. S2.1}$$

According to the Nernst equation, the solid phase and water are not included, giving the following equation:

$$E_{Mn(VII)/(IV)} = 1.70 - \frac{0.05916}{3} log\,(\frac{1}{[MnO^{-}_{4}][H^{+}]^{4}}) \qquad \textbf{Eq. S2.2}$$

$$E_{Mn(VII)/(IV)} = 1.70 + 0.0197\, log\,([MnO^{-}_{4}]) - 0.07888(pH) \qquad \textbf{Eq. S2.3}$$

Further reduction of Mn^{4+} to Mn^{3+} will be based on the reaction below: [2]

$$MnO_{2\,(s)} + 4H^{+} + e^{-} \leftrightarrow Mn^{3+} + 2H_2O \qquad E^o = 0.95\ V \qquad \textbf{Eq. S3}$$

Further, giving the Nernst equation of:

$$E_{Mn(IV)/(III)} = 0.95 - \frac{0.05916}{1} log\,(\frac{[Mn^{3+}]}{[H^{+}]^{4}}) \qquad \textbf{Eq. S3.1}$$

$$E_{Mn(IV)/(III)} = 0.95 - 0.05916\, log\,([Mn^{3+}]) - 0.2366(pH) \qquad \textbf{Eq. S3.2}$$

Based on the equation above, if our system gives a reduction from Mn^{7+} to Mn^{3+}, the reaction and the Nernst equation will be a combination of **Eq. S2** and **Eq. S3**, as follows:

$$MnO^{-}_{4\,(aq)} + 8H^{+} + 4e^{-} \leftrightarrow Mn^{3+} + 4H_2O \qquad \textbf{Eq. S4}$$

E^o Mn(VII)/(III) = $\frac{(n1 \times E^o Mn^{7+/4+}) + (n2 \times Mn^{4+/3+})}{n1+n2}$

= $\frac{(3\times1.70)+(1\times0.95)}{4}$

= 1.5125 V

$$E_{Mn(VII)/(III)} = 1.5125 - \frac{0.05916}{4} log\,(\frac{[Mn^{3+}]}{[MnO^{-}_{4}]\,[H^{+}]^{8}}) \qquad \textbf{Eq. S4.1}$$

$$E_{Mn(VII)/(III)} = 1.5125 - 0.0148\,(log\,([Mn^{3+}]) - log\,([MnO^{-}_{4}])) - 0.11832(pH)$$

Eq. S4.2

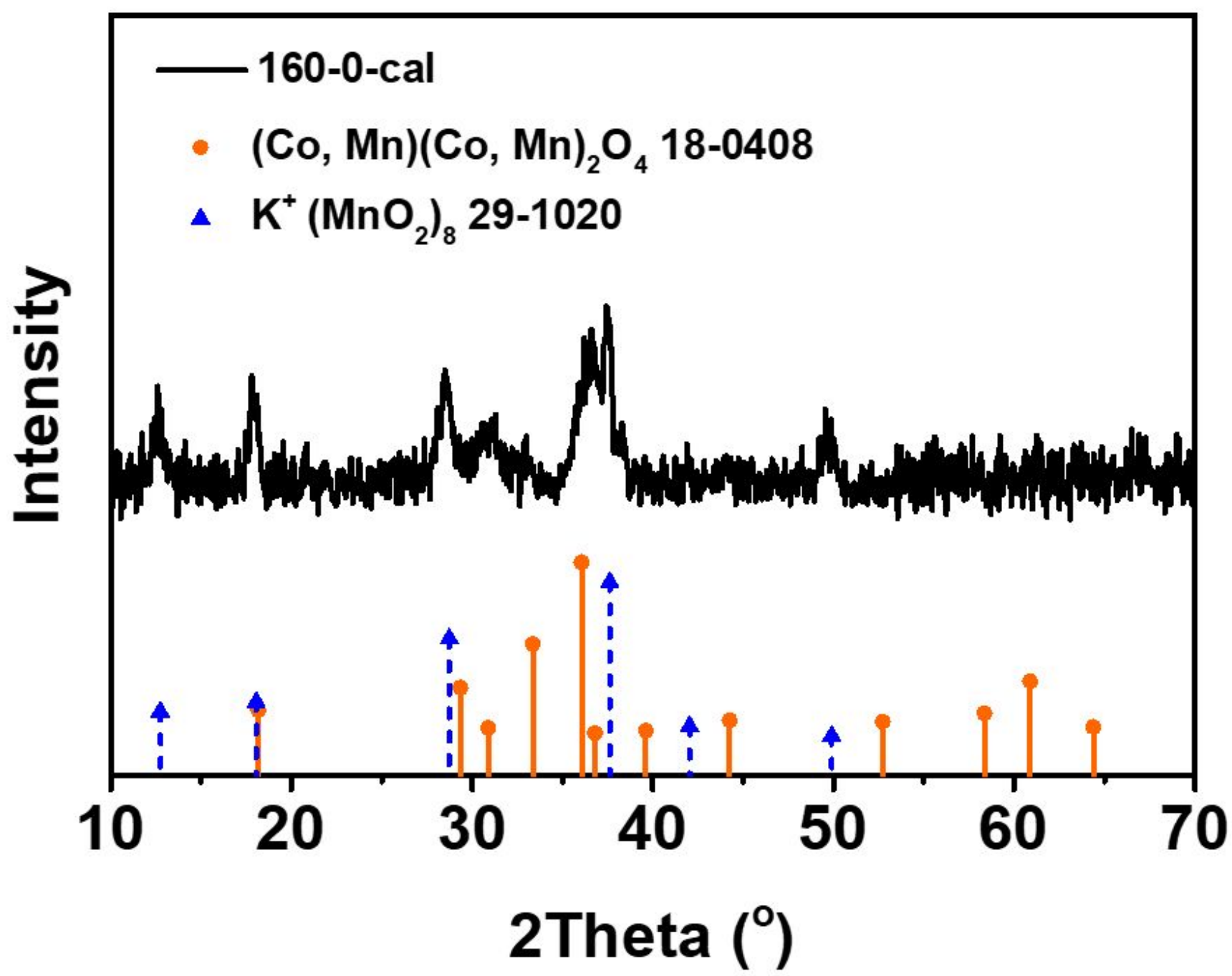

Figure S1. XRD pattern of the 160–0-cal, matched with reference peak positions of tetragonal spinel (Co, Mn) (Co, Mn)$_2$O$_4$ (JCPDS 18-0408) and tunnel-type hollandite K$^+$(MnO$_2$)$_8$ (JCPDS 29-1020).

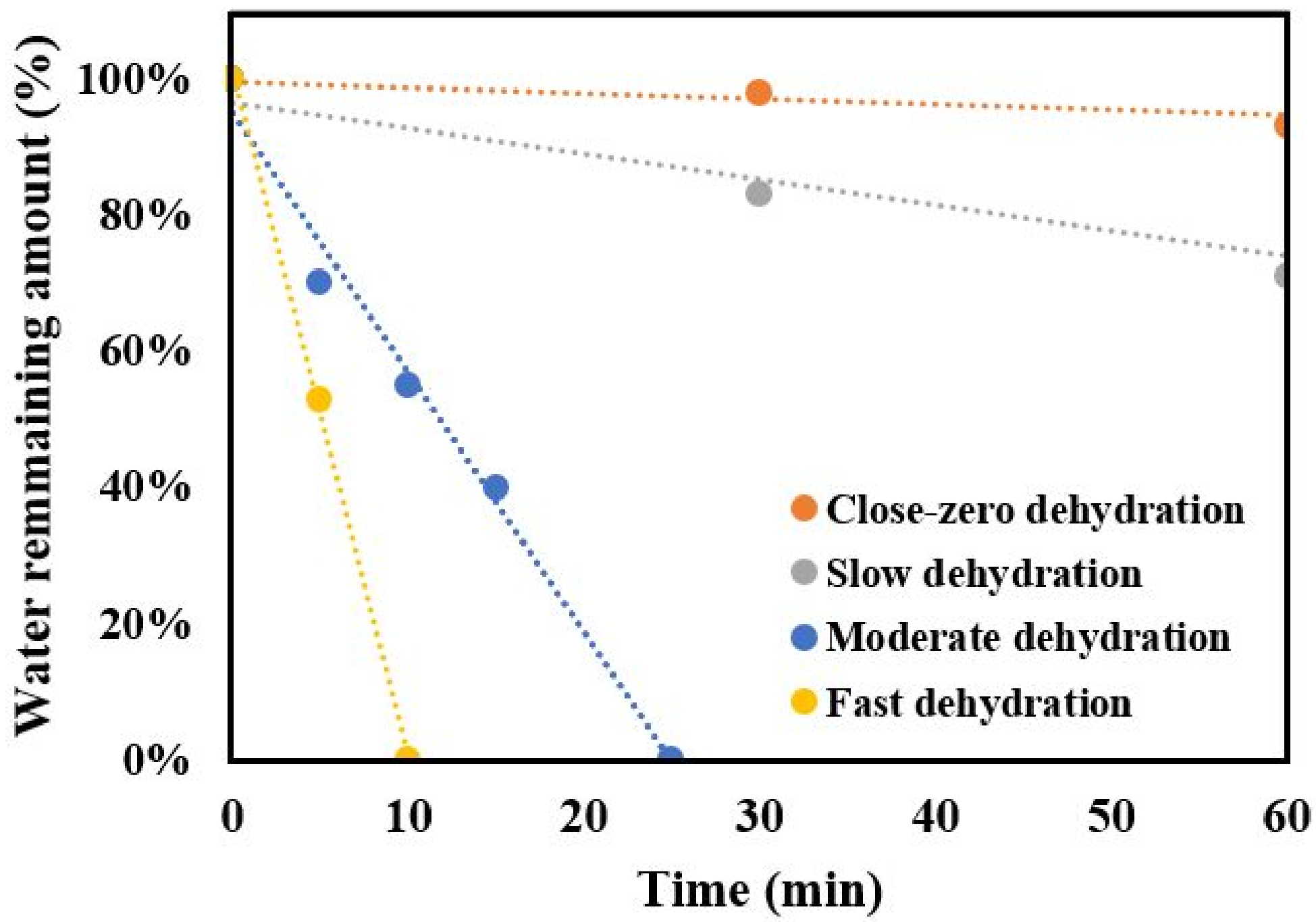

Figure S2. Remaining water percentage for a blank system at 160°C containing 1.0 mL deionized water under different dehydration condition.

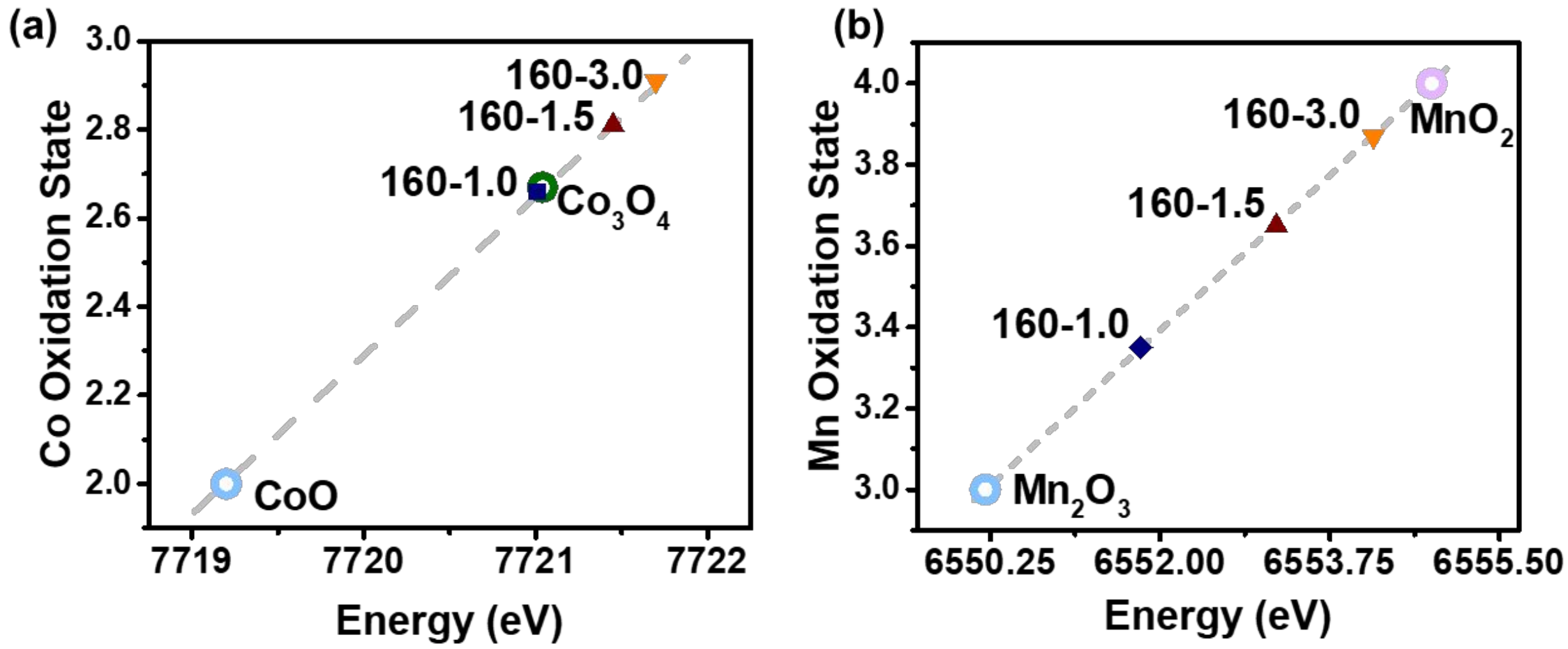

Figure S3. The corresponding oxidation number based on the linear fitting of the absorption edge position of XANES spectra of (a) Co K-edge and (b) Mn K-edge.

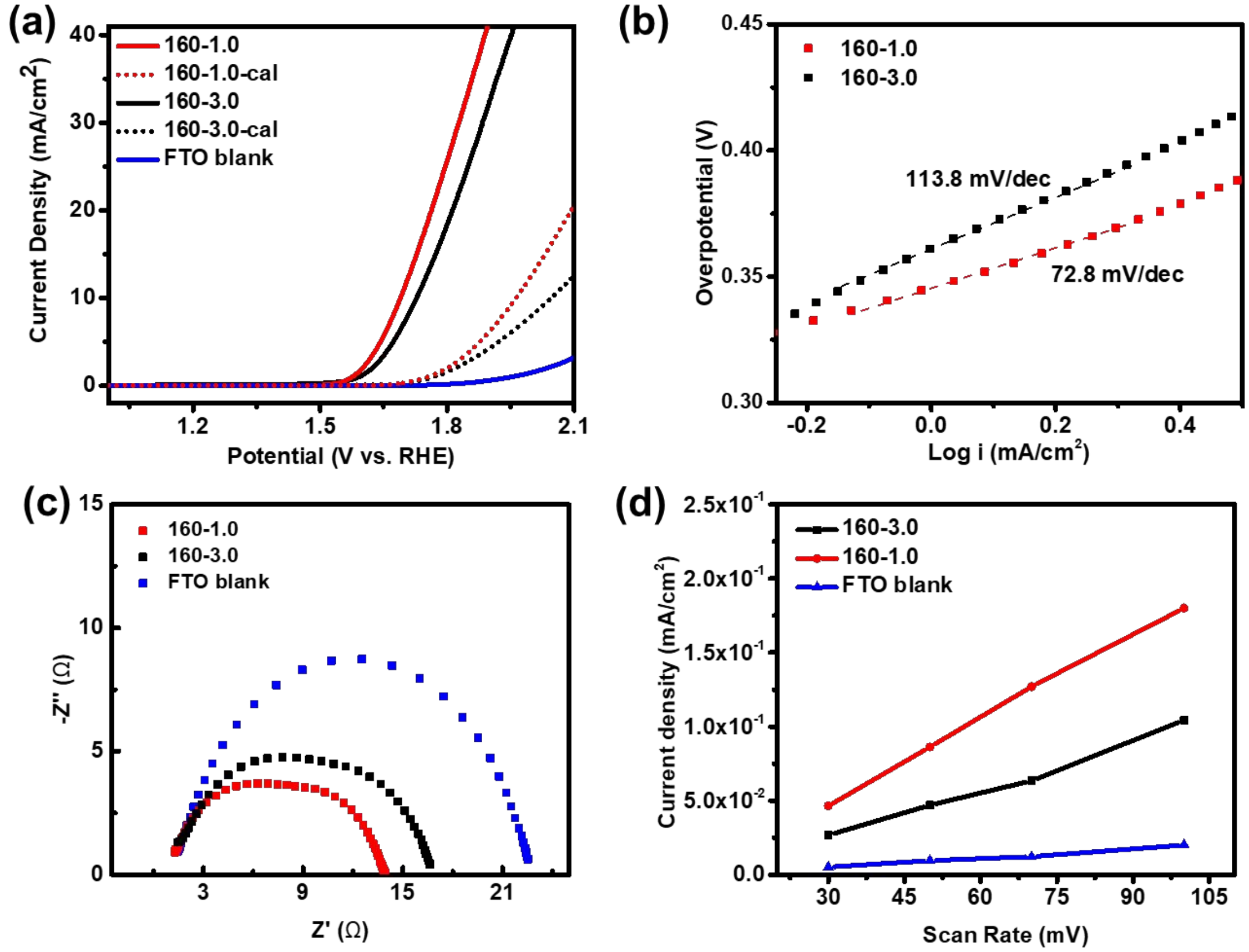

Figure S4. The comparison of electrochemical performance between LRS system samples with tetragonal and cubic spinel structures. (a) The polarization curves for 160-1.0, 160-1.0-cal, 160-3.0, and 160-3.0-cal and the FTO blank (substrate) reference in 1.0 M KOH at 5 mV s^{-1}, and (b) corresponding Tafel plots for 160-1.0 and 160-3.0. (c) The EIS Nyquist plots (d) The current density versus scan rate under the non-faradaic region for C_{dl} determination for 160-1.0 and 160-3.0.

Table S1. Initial water addition and corresponding liquid-to-solid ratios (η) used during the liquid-assisted redox synthesis of Co–Mn oxides.

Sample	Water (mL)	η (μL mg^{-1})
160–0.5	0.5	0.25
160–1.0	1.0	0.50
160–1.5	1.5	0.75
160–3.0	3.0	1.50
160–5.0	5.0	2.49
160–10.0	10.0	4.99

Table S2. Quantitative assessment of residual water under different dehydration conditions after 2 h heating at 160 °C.

**Dehydration condition**	**Dehydration environment**	**Remaining volume (mL)[a]**	**Water removal rate (mL/min $\times$ 10^{-3})**	**Any residual water? (wt%)[b]**
Close-zero (cd)	Teflon autoclave or quasi-sealed	0.905 ± 0.021	0.8	Yes (90.5 ± 2.1)
Slow (sd)	Sealed crucible	0.536 ± 0.082	0.4	Yes (53.6 ± 8.2)
Moderate (md)	Crucible + lid	N. A.	40	No (N. A.)
Fast (fd)	Crucible without lid	N. A.	100	No (N. A.)

[a] Remaining volume of water was measured as mass (g) then convert to volume using the density of water (1 mL ≈ 1 g)
[b] Residual water (wt%) = (volume of remaining water / initial volume of water) × 100.

Table S3. The Co:Mn composition ratios determined by ICP analysis for Co–Mn oxide samples synthesized with different LRS initial water environment.

Sample	Co:Mn Ratio (ICP)	Phase (XRD)
160-0.5	2.10 ± 0.08 :1	Tetragonal
160-1.0	2.17 ± 0.10 : 1	Tetragonal
160-1.5	1.91 ± 0.13 : 1	Tetragonal
160-3.0	2.30 ± 0.20 : 1	Cubic
160-5.0	2.55 ± 0.17 : 1	Cubic
160-10.0	2.90 ± 0.10 : 1	Cubic
Conventional ARP (150 mL)[2]	3.05 ± 0.10 : 1	Cubic

Table S4. The effect of the amount of water added on the product yield for the LRS system method.

Samples	Mass product (g)	Percent Yield
160-0.5	0.452 ± 0.015	80.3 ± 2.7%
**160-1.0**	**0.486 ± 0.017**	**86.3 ± 3.0%**
160-1.5	0.402 ± 0.013	71.4 ± 2.4%
160-3.0	0.094 ± 0.025	16.7 ± 4.4%
160-5.0	0.086 ± 0.022	15.3 ± 4.0%
160-10.0	0.088 ± 0.020	15.6 ± 3.6%
Conventional ARP (150 mL)[2]	0.018 ± 0.010	3.2 ± 1.8%
160-1.0-fast	0.483 ± 0.022	85.7% ± 4.0%
160-1.0-moderate	**0.486 ± 0.017**	**86.3 ± 3.0%**
160-1.0-slow	0.456 ± 0.028	81.0 ± 5%
160-1.0-close-zero	0.435 ± 0.025	77.6 ± 4.4%

*All yield measurements were obtained from three independent synthesis batches (n = 3) and are reported as mean ± standard deviation.

Table S5. The double-layer capacitance (C_{dl}) and electrochemically active surface area (ECSA) results for the uncalcined LRS catalyst system.

**Samples**	**C_{dl} (μF/cm^2)**	**ECSA**
FTO blank	2.8	1
160-3.0	10.5	3.75
160-1.0	18.9	6.75

References

1. Bratsch, S. G., Standard Electrode Potentials and Temperature Coefficients in Water at 298.15 K. *J. Phys. Chem. Ref. Data* **1989,** *18*, 1-21.
2. Jhang, R.-H.; Yang, C.-Y.; Shih, M.-C.; Ho, J.-Q.; Tsai, Y.-T.; Chen, C.-H., Redox-assisted multicomponent deposition of ultrathin amorphous metal oxides on arbitrary substrates: highly durable cobalt manganese oxyhydroxide for efficient oxygen evolution. *J. Mater. Chem. A* **2018,** *6*, 17915-17928.
